# Medicinal Uses, Phytochemistry, and Pharmacological Activities of *Quercus* Species

**DOI:** 10.1155/2020/1920683

**Published:** 2020-07-31

**Authors:** Mehdi Taib, Yassine Rezzak, Lahboub Bouyazza, Badiaa Lyoussi

**Affiliations:** ^1^Laboratory of Renewable Energy, Environment and Development, Hassan 1st University Faculty of Science and Technology, P.O. Box 577, Settat, Morocco; ^2^Laboratory of Natural Substances, Pharmacology, Environment, Modeling, Health and Quality of Life (SNAMOPEQ), University of Sidi Mohamed Ben Abdellah, Fez 30 000, Morocco

## Abstract

*Quercus* species, also known as oak, represent an important genus of the Fagaceae family. It is widely distributed in temperate forests of the northern hemisphere and tropical climatic areas. Many of its members have been used in traditional medicine to treat and prevent various human disorders such as asthma, hemorrhoid, diarrhea, gastric ulcers, and wound healing. The multiple biological activities including anti-inflammatory, antibacterial, hepatoprotective, antidiabetic, anticancer, gastroprotective, antioxidant, and cytotoxic activities have been ascribed to the presence of bioactive compounds such as triterpenoids, phenolic acids, and flavonoids. This paper aimed to provide available information on the medicinal uses, phytochemicals, and pharmacology of species from *Quercus*. However, further investigation is needed to fully clarify the mode of action of its bioactive compounds and to evaluate *in vivo* chronic toxicity, before exploring their potential use as a supplement in functional foods and natural pharmaceutics.

## 1. Introduction

Since primitive times, humans have been using plants for their essential requirements such as food and medicine. These plants have been used in traditional medicine in order to cure and prevent various human disorders. The important advantage for therapeutic uses of the plants includes their safety, effectiveness, economic feasibility, and ease of availability [1]. Recently, the global demand for medicinal plant products has increased from USD 19.6 billion in 1996 to USD 24.2 billion in 2002 and is projected to reach USD 5 trillion by 2050 [2]. Among a number of medicinal plants, species belonging to the genus *Quercus* are widely used in traditional medicine. This genus belongs to the family Fagaceae. It comprises 600 species worldwide, which often differ in their flowering and fruiting dynamics and by the maturation index [3]. Species of the *Quercus* genus are mainly distributed in the basin Mediterranean (Portugal, Spain, Algeria France, Italy, Tunisia, and Morocco), Asia, and North America. The extraordinary species diversity reported in America and Asia together is with the highest diversity at 15–30°N in Mexico and East Asia [4, 5]. Europe exhibits lower species richness (up to 30 species), but the genus is nearly as widespread there as it is in North America and East Asia, as a limited number of European species have expanded across the continent [6].

Species of the genus *Quercus* are important medicinal plants. Over the centuries, these species have been used in folk medicine to treat various diseases Table 1. Indigenous peoples, in many areas of the world, use them as antiseptics and to treat gastrointestinal tract (GIT) disorders such as diarrhea and hemorrhoids. The bark of the oak has much importance and is used extensively in medicine as an antiseptic and hemostatic, used to cure toothache and gastropathies, and also used as pacifying agents in inflammation and as healing agents in burn [34, 37, 38]. However, the resin of *Quercus leucotrichophora A. Camus* is used to cure gonorrhea, asthma, hemorrhages, diarrhea, and dysentery [34]. Powder of gallnuts of *Q. infectoria* is used to restore the elasticity of the uterine wall, as well as to treat aphthous ulcers [31, 39]. The fruit (acorn) of the *Quercus* species is considered as a nutritionally rich source of energy (source of carbohydrates, proteins, and fat), justifying their use as food or ingredient food for thousands of years in the human diet such as in bread production or as an ingredient for making coffee [40–42]. The acorns of the various species of oak are widely used in curing diarrhea, laryngopharyngitis diseases, menorrhagia, obesity, and stomach ulcers [8, 9].

Pharmacological effect reported for the *Quercus* genus plant includes antioxidant, antimicrobial, anti-inflammatory, antidiabetic, hepatoprotective, gastrointestinal disorder, skin disorder, antiobesity, anticancer, and neurogenerative effect [43–49]. All mentioned effects are attributed to the specific chemical composition, comprised mainly of triterpenoids, flavonoids, and tannins. As an example, Endo et al. [50] investigated the antitoxoplasma effect of the *Quercus crispula Blume* outer bark. The authors identified three pentacyclic triterpenoids, namely, 29-norlupane-3,20-dione, oleanolic acid acetate, and ursolic acid acetate, and concluded that these compounds exhibited notable activities against the *Toxoplasma gondii* parasite. Moreover, Lei et al. [45] successfully isolated new triterpenoids, which were identified as ursane, oleanane, and lupinane type and were found to be associated with the antineuroinflammatory activity. Even so, numerous studies describing the bioactivities of acorns are focused on their strong antioxidant activity, which are believed to be useful in treating oxidation-associated diseases such as diabetes, cancer, and cardiovascular and inflammatory diseases [41, 51–53].

The purpose of this review is to provide up-to-date information on traditional medicinal uses, phytochemistry, and pharmacological activities of *Quercus* species in order to explore their therapeutic potential and evaluate future research opportunities.

## 2. *Quercus* Genus

The oak family (*Quercus* spp.) plays a major ecological role in terms of sheer abundance of standing biomass [54]. The genus *Quercus* is among the most widespread and species-rich tree genera in the northern hemisphere [6]. The highest diversity is exhibited in Mexico and East Asia [4, 5]. Europe exhibits lower species richness (up to 30 species), but the genus is nearly as widespread there as it is in North America and East Asia, as a limited number of European species have expanded across the continent [6]. *Quercus* spp. (oak) represent an important genus of the Fagaceae family which consists of 600 species worldwide, which includes monecious, deciduous, evergreen trees, and rarely shrubs. The genus *Quercus* has long been considered one of the most imperative clades of all woody plants in terms of species diversity, ecological dominance, and economic value. The leaves of many oak species are conspicuously lobed, but some species reveal variations in shape from small to large and pointed. Oaks are considered monecious plants, having separate male and female flowers on a single tree. Generally, the male flowers occur in clusters but sometimes are organized in a form called catkin. The female flowers are borne on solitary spikes in the axils of leaves or bracteoles of the new growth [55]. The flowers mostly ripen in the sepals, which later mature into the fruit. The fruit identified as acorn, which is a nut, is characterized by the absence of an endosperm and the presence of an achlorophyllous embryo.

## 3. Medicinal Uses of Some Species from *Quercus*

*Quercus* species have long been used as traditional medicine in several countries and tribes. Almost all parts of the plants including fruit, bark, and leaves were documented to display a broad range of medicinal properties (Table 1). Indigenous peoples, in many areas of the world use them as antiseptics and to treat gastrointestinal tract (GIT) disorders such as diarrhea and hemorrhoids (Table 1). It is widely used to treat gonorrhea, gastritis, asthma, pyrexia, Parkinson's disease, and hepatoprotective diseases [56], and also hemorrhoid, varicose veins, diarrhea, gastric ulcers, and superficial injuries [57].

The bark of the oak has much importance and is used extensively in medicine as an antiseptic and an energizer. The decoctions from the barks of *Q. robur* and *Q. petraea* were recognized to have anti-inflammatory, antibacterial, and antihemorrhagic activities [58]. Indeed, it has been suggested for the treatment of patients with high levels of blood sugar [59] and treatment of sore throat [24]. The leaves of *Q. virginiana* have been used as antimicrobial agent. As well as, can be employed for the treatment of gastrointestinal disorders [60, 61]. The galls of *Q. infectoria* are used to restore the elasticity of the uterine wall, as well as to treat many inflammatory disorders [31]. Also, they are used in Malay traditional medicine commonly to treat wound infections after childbirth [32]. In India, they are employed traditionally as dental applications such as that in treatment of toothache and gingivitis. In Asia, it has been widely used for the treatment of infection diseases, skin disorders, and inflammatory ailments [39]. The traditional system of medicine is an integral part of Kumaun and Garhwal regions of Uttarakhand, and others states in India used *Quercus leucotrichophora A. Camus* for treatment of urinary infection [34], stomach pain [35], gonorrhea, asthma, hemorrhages, diarrhea, dysentery [33], urinary disorder [62], and diuretics [63]. Besides, in Korean medicine folk, they are widely used for their dysentery, antidiarrhea, and antidermatitic proprieties [64].

The fruit (acorn) of oak species is a rich source of energy, containing high amounts of carbohydrates, proteins, amino acids, lipids, and sterols. The earliest evidence of acorns as a foodstuff was dated to the late Mesolithic era and found in Western Europe. In the North American West Coast, acorns made up more than half of the diet of native peoples [65]. The fruit from *Quercus humilis Mill* are mainly consumed roasted, raw, or as an ingredient for making coffee only in particular areas [40]. In the northeast of the Iberian Peninsula, the fruit of holm oak *Q. ilex* subsp. locally known as kiskurras were used raw, boiled, roasted, like coffee, or transformed into flour. In addition, its flour was mixed with cereal grain flour to make bread [66]. Similarly, in Serbia, *Quercus cerris* seeds are widely used for bread production [16]. Also, the oil obtained from the acorn has been consumed by native peoples for hundreds of years. It is a nutritious cooking oil comparable to those obtained from peanut, cotton, olive, and avocado [52, 65].

## 4. Phytochemicals

The *Quercus* genus contains various classes of compounds such as glycosides, terpenoids, flavonoids, phenolic acids, fatty acids, sterols, and tannins. The polyphenols isolated from the *Quercus* genus are reported in Table 2. Despite the phylogenetic variability, phenolic acids (particularly, gallic and ellagic acids and their derivatives), flavonoids (particularly flavan-3-ol), and tannins are abundant in all the species of *Quercus* [52]. To date, seven compounds were isolated from *Quercus gilva Blume* (QGB) and identified as picraquassioside D, quercussioside, (+)-lyoniresinol-90*α*-*O*-*β*-D-xylopyranoside, (+)-catechin, (−)-epicatechin, procyanidin B3, and procyanidin B4. The presence of these compounds suggests that QGB could be used in the treatment of urolithiasis [80]. In addition, Gul et al. [78] successfully isolated a new compound, quercuschin, with six other compounds which were identified as quercetin, methyl gallate, gallic acid, betulinic acid, (Z)-9-octadecenoic acid methyl ester, and *β*-sitosterol glucoside from *Q. incana*. Indeed, the compounds such as eupatorin (5,3′-dihydroxy-6,7,4′ trimethoxyflavone), cirsimaritin (4′,5, -dihydroxy-6,7-dimethoxyflavone), betulin (lup-20(29)-ene-3, 28-diol), and *β*-amyrin acetate (12-oleanen-3yl acetate) were also observed in the leaves of *Quercus incana* [29].

The main compound found in the methanol leaves extract of *Quercus suber* was gentisic acid [84]. However, another study performed by Yin et al. [85], who analyzed the 50% ethanol crude extract of *Mongolian oak cups*, demonstrated that ellagic acid (EA) and kaempferol derivatives were the main phenolic components. Moreover, Sari et al. [83] reported five compounds, which are lyoniresinol-9-*O*-*β*-xylopyranoside, lyoniresinol- 9-*O*-*β*-glucopyranoside, (-)-8-chlorocatechin, polydatin, and cocciferoside that occur in the *Quercus coccifera* bark.

Using advanced spectroscopic techniques, Indrianingsih et al. [86] isolated catechin, epicatechin, and tiliroside from *Q. gilva*. The tiliroside found in *Q. gilva* can inhibit *α*-glucosidase activity. Flavonoids are also found to be important bioactive compounds of *Quercus* leaves. Xu et al. [87] conducted a study on acorn leaves (*Quercus liaotungensis*) and isolated one new flavonoid glycoside, namely, 2,3-diol acetonide-3-*O*-*α*-L-rhamnopyranoside-kaempferol, and 25 known polyphenolics. Recently, a study carried out on the infusion of the leaves of the six different species of *Quercus* revealed the presence of 7 flavan-3-ols, 2 flavonols, 18 flavonols/flavanone glycosides, 1 flavanone, 1 flavone, and 4 other unknown compounds. In this context, the presence of 4 procyanidin dimers in infusions was also documented [79].

**Figure 1:**
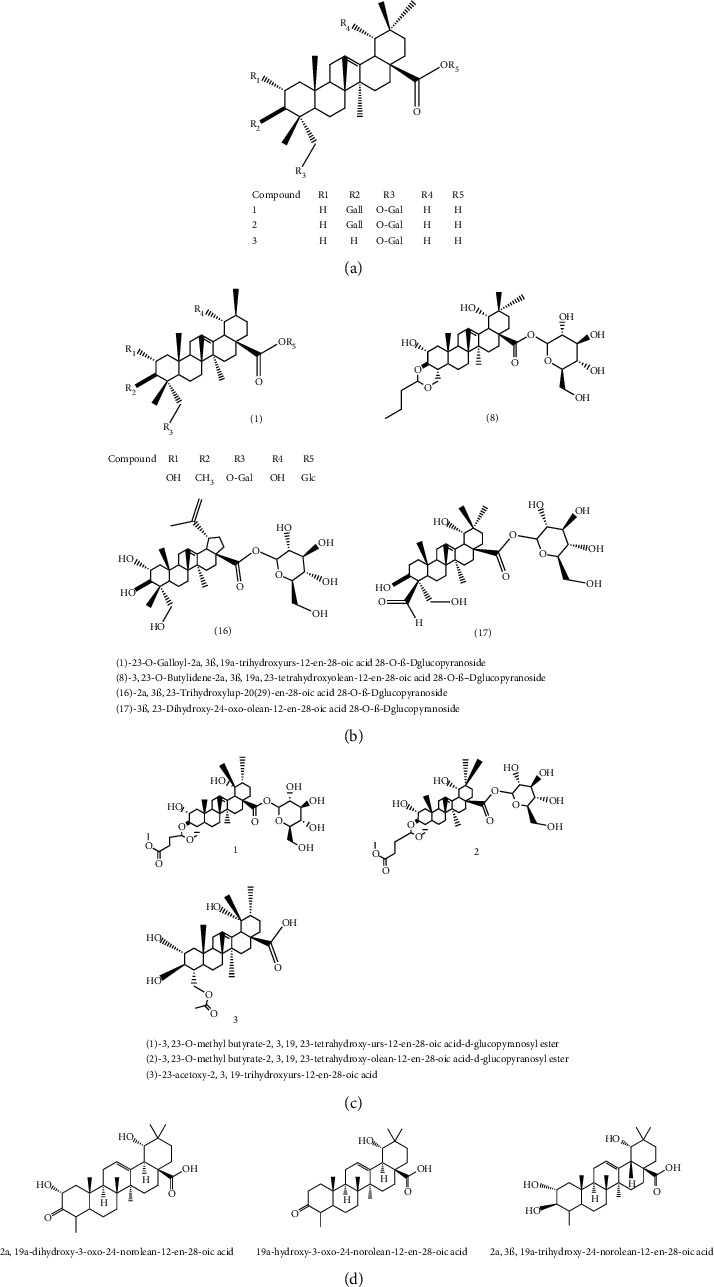
(a) Chemical structure of the 3 new pentacyclic triterpenes which were elucidated to be 3-O-galloyloleanolic acid, 23-acetoxy-3-O-galloyloleanolic acid, and 3-acetoxy-23-O-galloyloleanolic acid from the *Q. liaotungensis* acorn [49]. (b) Four new triterpenoid saponins from *Q. serrata var. brevipetiolata* [45]. (c) New triterpenoid saponins isolated from acorns of *Q. serrata var. brevipetio*lata [44]. (d) Three new 24-noroleanane triterpenoids from *Q. aliena var. acuteserrata* [88].

Triterpenoids have also been isolated from the species of *Quercus*. Recently, Xu et al. [87] have identified 3 new pentacyclic triterpenes which were elucidated to be 3-O-galloyloleanolic acid, 23-acetoxy-3-O-galloyloleanolic acid, and 3-acetoxy-23-O-galloyloleanolic acid (Figure 1(a)), along with 22 compounds known from the *Q. liaoningensis* acorn which showed antidiabetic effect. Lei et al. [45] successfully isolated new triterpenoids which were identified as ursane, oleanane, and lupinane type and were found to be associated with the antineuroinflammatory activity (Figure 1(b)). In another study, ten pentacyclic triterpenes, three of which were novel, were isolated from acorns *Quercus serrata var. brevipetiolata* [44] (shown in Figure 1(c)).

**Figure 2:**
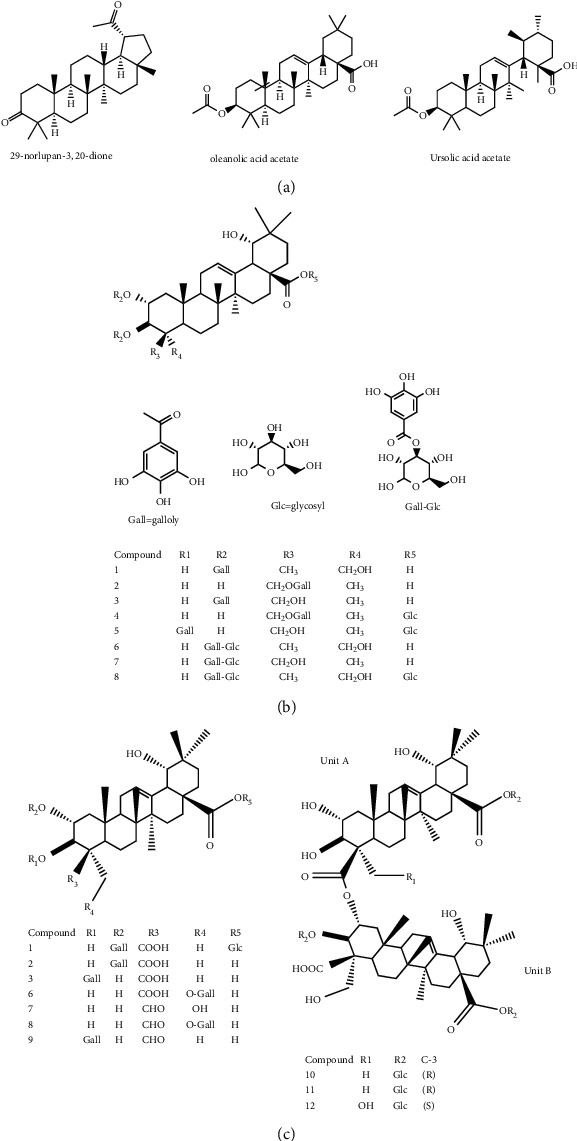
(a) Chemical structure of three pentacyclic triterpenoids isolated from the *Q. crispula Blume* outer bark [50]. (b) Eight new triterpenoids (1−8) isolated from *Q. petraea* [89]. (c) 12 new triterpenoids identified from oak heartwood *Q. robur* [90].

Endo et al. [50] investigated the antitoxoplasma effect of the *Quercus crispula Blume* outer bark. The authors identified three pentacyclic triterpenoids, namely, 29-norlupane-3,20-dione, oleanolic acid acetate, and ursolic acid acetate (Figure 2(a)) and concluded that these compounds exhibited notable activities against the *Toxoplasma gondii* parasite. Using *HRESIMS* and *1D/2D* NMR experiments, Gammacurta et al. [89] screened EtOAc extracts from the *Quercus petraea* heartwood for phytochemical investigations and isolated eight new triterpenoids (1−8) (Figure 2(b)) and two known functionalized triterpenoids. Posteriorly, Perez et al. [90] in their study, identified 12 new triterpenoids, 1−12 (Figure 2(c)), and five known oleanane types which showed cytotoxicity activity against cancer cells (PC3 and MCF-7) and lymphocytes.

**Figure 3:**
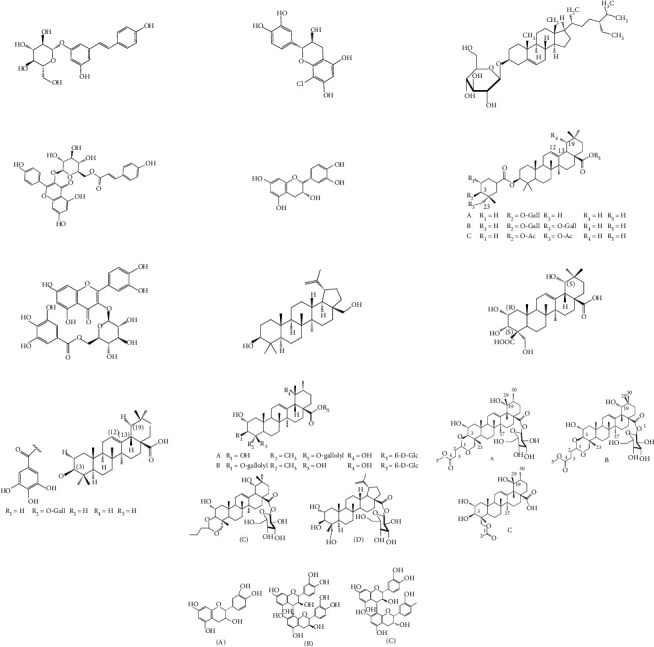
Chemical structures of bioactive compounds of *Quercus* species.

Previously, three new 24-noroleanane triterpenoids, 2a,19a-dihydroxy-3-oxo-24-norolean-12- en-28-oic acid, 19a-hydroxy-3-oxo-24-norolean-12-en-28-oic acid, and 2a,3b,19a-trihydroxy-24-norolean-12-en-28-oic acid (Figure 1(d)), were also isolated from *Q. aliena* var. *acuteserrata*, with previously known compounds (bartogenic acid, ilexgenin, and aophitolic acid) [88]. The chemical structures of the bioactive compounds of *Quercus* species are shown in Figure 3.

## 5. Pharmacological Activities

Traditional uses of *Quercus* species have led researchers to investigate their biological activities and to validate the uses of species of the genus as therapeutic remedies. Several pharmacological activities have been reported to be exhibited by extracts as well as single compounds, such as antioxidant, antibacterial, anti-inflammatory, and cytotoxicity activity.  Table 3 summarizes the major bioactive compounds of *Quercus* species and its pharmacological activities.

### 5.1. Antioxidant Activity

*Quercus* genus has been reported to possess antioxidant activity [41, 42, 69, 92]. A recent study by Arina and Harisun [93] has evaluated the effect of extraction temperatures on the tannin content and antioxidant activity of *Quercus infectoria* (*Manjakani)*. According to the result, the extract gives high DPPH scavenging capacity with an IC50 value of 0.064 mg/ml at the extraction temperature of 75°C. Another study showed that the thermotreatment and extraction technique had a determinant role in the antioxidant efficiency of *Quercus cerris* L. wood [94]. The antioxidant activities of leaves and acorn of *Q. suber* were investigated using 3 different solvents (hexane, methanol, and water). In this case, the aqueous extracts displayed the highest antioxidant activity, based on DPPH and ABTS assays. This antioxidant activity might be ascribed to the presence of phytochemical compounds such as phenolic compounds in the acorn extracts [69, 92].

*Q. sideroxyla* infusions have exhibited the highest antioxidant activity followed by *Q. eduardii* and *Q. durifolia* infusions, based on Trolox equivalent antioxidant capacity [95]. In addition, *β*-sitosterol-D-glucoside and condensed tannin fractions **(2, 3, 4, 5, 6)** isolated from the leaves of *Quercus phillyraeoides* have also been studied for their antioxidant potential. The highest DPPH scavenging capacity was exhibited by fraction **5**, followed by fractions **3**, **2**, **6**, and **4** with IC50 values of 9.34, 10.53, 10.84, 12.98, and 13.12 *μ*g/ml, respectively [91]. Furthermore, Amessis-Ouchemoukh et al. [96] investigated the antioxidant activity of carob pods (*Ceratonia siliqua*), white figs (*Ficus carica*), and acorn (*Quercus ilex*). Their results showed that *Quercus ilex* and *Ceratonia siliqua* were very effective in scavenging DPPH and ABTS radicals, 93.93 ± 0.13 and 82.45 ± 0.23% in the DPPH assay and 83.09 ± 0.07 and 81.51 ± 0.12% in the ABTS assay, respectively. As expected in this research, the obtained inhibitions were better than those displayed by standards BHA, catechin, quercetin, and trolox with 26.63 ± 0.56, 56.09 ± 0.24, 70.43 ± 0.15, and 61.21 ± 1.15%, respectively. Extracts from other *Quercus* species have been also tested. As an example, Sánchez-Burgos et al. [46] showed that aqueous extracts obtained from the leaves of different white *Quercus* species (*Q. grisea, Q. laeta, Q. obtusata, and Q. resinosa*) exhibited high radical scavenging activity against (DPPH) and HO• radicals.

Makhlouf et al. [97] performed antioxidant activity analysis of fixed oil from three acorn species grown in Algeria: *Quercus ilex* L, *Quercus suber* L, and *Quercus coccifera* L and observed that oils methanolic extracts had remarkable antioxidant activity, up to 3.34 and 3.79 *µ*mol TE g−1 oil in the DPPH and ABTS tests, respectively.

Reported findings from different scientists illustrate that *Quercus* species are a good source of natural antioxidants which can be explored as ingredients for functional food and nutraceutical industry.

### 5.2. Antibacterial Activity

The antibacterial activity of *Quercus* has been investigated against Gram-positive and Gram-negative bacteria including multidrug-resistant bacterial pathogens. An aqueous extract of leaves from four species of white oaks (*Q resinosa, Q laeta, Q grisea, and Q obtusata*) was investigated for antimicrobial activities against a range of bacteria (*E. coli*, *S. epidermidis*, *K. pneumoniae*, *P. mirabilis*, *P. hauseri*, *P. vulgaris*, and *E. aerogenes*) and yeast (*C. albicans*). They further reported that all aqueous extracts of oak tested showed susceptibility to *K. pneumoniae* (*ATCC 13883*). These researchers also reported that *Q. resinosa* and *Q. grisea* denoted antimitotic activity against these organisms [46], whereas Bahador and Baserisalehi [98] tested the antibacterial activity against Gram-negative bacteria (*E. coli, Salmonella typhimurium, Shigella dysenteriae, and Yersinia enterocolitica*) of the fruit of *Q. castaneifolia*. According to their finding, *S. dysenteriae* was more sensitive with a zone of inhibition of 18 mm, and he MIC value was 2.5 × 10^−4^. The lowest MIC values were found for extracts for *E. coli*. In a study carried out by Sarwar et al. [28], the antibacterial activity of the gold nanoparticle synthesized from the leaves of *Quercus incana* was evaluated against the human pathogens (*Pseudomonas pickettii, Salmonella setubal, Staphylococcus aureus, Bacillus subtilis, Aspergillus flavus,* and *Aspergillus niger*). The results showed enhanced antibacterial activity against all bacterial pathogens. Besides, the ethanolic extracts of *Q. persica* have been also tested against *S. aureus, B. subtilis, E. coli, and K. pneumoniae* [99].

Hobby et al. [100] tested the ability to inhibit the *Staphylococcus aureus* biofilm using the leaf, stem, and fruit from *Quercus cerris.* The activity was measured using static crystal violet staining methods and confocal laser scanning microscopy. The study revealed that butanol extracts of both the leaf and stem/fruit samples were the most active, at a dose of 200 ug/ml.

In an agar-well diffusion assay, the methanol and acetone extracts of the gall of *Q. infectoria* showed activity against oral pathogens such as *Streptococcus mutans ATCC 25175, Porphyromonas gingivalis ATCC 33277, Streptococcus salivarius ATCC 13419*, and *Fusobacterium nucleatum ATCC 25586*. The MIC ranged from 0.16 to 0.63 mg/mL, and the most susceptible bacterium was *S. salivarius,* which suggested that the oak extract might be used against dental caries and periodontitis etiological agents [101]. In another study, the extracts of *Quercus infectoria* were assessed against many microbial species and used for eggshell decontamination. The antimicrobial activity was evidenced against *Staphylococcus aureus, Escherichia coli, Pseudomonas aeruginosa, Salmonella typhimurium*, and *Candida albicans*, and the results showed disinfection of eggshell microbial contamination, by immersion in 1% *QIE* solution, sharply reduced total colony count, yeasts, and molds, and Enterobacteriaceae, *E. coli*, *and S. aureus* were completely inhibited after 60 min of immersion in QIE [102]. Another investigation demonstrated that the extract of the *Quercus infectoria* gall possesses antimicrobial activity against *Leptospira interrogans serovar Javanica* and *Leptospira interrogans serovar Icterohaemorrhagiae* with MIC values of 0.125 mg/mL [103].

In another work, Touati et al. [71] tested the antibacterial activity of the cork from *Quercus suber* L against *Staphylococcus aureus ATCC, Listeria innocua, Escherichia coli*, and *Pseudomonas aeruginosa*. The plant was collected from Algeria. The phenolic fraction of the cork was shown to inhibit the growth of *S. aureus* (12.1 mm) and *P. aeruginosa* (10.07 mm).

Therefore, different types of *Quercus* can be used as an alternative source of potential antimicrobial agents, and more analysis in *in vivo* and clinical studies is required to substantiate these *in vitro* findings.

### 5.3. Cytotoxic and Anticancer Activity

Several studies have confirmed the cytotoxic and anticancer activity of a wide variety of *Quercus* species extracts, against various cancer cell lines. The MeOH and water extracts of the barks of *Quercus cerris* var. *cerris, Quercus macranthera* subsp. *syspirensis*, and *Quercus aucheri* were subjected to the evaluation of their cytotoxicity against the Hep-2 human larynx epidermoid carcinoma cell line. The results demonstrated that aqueous and methanolic extracts of *Q. macranthera* subsp. *syspirensis* showed the strongest cytotoxicity against the tested cell line, with IC50 values 165.291 ug/ml and 273.771 ug/ml, respectively [104]. In addition, the ethanolic extract of *Quercus ilex* has been studied for its cytotoxicity by the MTT assay in various concentrations (250, 500, and 1000 mg/mL). The results indicated that the treatment inhibited cell viability in a dose- and time-dependent manner [96].

Perez et al. [90] studied the cytotoxic activity of 17 triterpenoids isolated from oak heartwood of *Quercus robur* against human prostate cancer (PC3) and human estrogen-dependent breast adenocarcinoma (MCF-7) cell lines and lymphocytes derived from human peripheral blood. The obtained results demonstrate that breast cancer cells (MCF-7) were the most affected by triterpenoids, with roburgenic acid, being the most active compound (IC50 = 19.7 *μ*M). The authors also reported the selectivity for some triterpenoids against lymphocytes, exhibiting an IC50 > 200 *μ*M, while active against cancer cells. Moreover, the genotoxicity of *Q. resinosa* leaves extracts was evaluated on HeLa cells by the single-cell electrophoresis assay (comet assay), indicating that phytochemical compounds present in extracts obtained from their decoctions increase the oxidative process and other damage to DNA in transformed human cells [47].

Recently, apoptotic and antimetastatic activities of betulin isolated from *Quercus incana* leaves were investigated against non-small-cell lung cancer. The results indicted significant dose-dependent induction of apoptosis after the treatment with betulin, followed by increased expression of the caspase family (i.e., caspase-3, -6, and -9), proapoptotic genes (BAX and BAK), and inhibiting antiapoptotic genes (BCL-2L1 and p53). Additionally, betulin was found to be highly and selectively active against the cancer cells at much lower doses (11.55 *μ*M) [29]. Also, it has been reported that the *Quercus suber* L. cork extracts induce apoptosis in human myeloid leukaemia HL-60 cells. The extracts showed a time-dependent and dose-dependent cytotoxicity in the human promyelocytic leukaemia cells [105].

Yarani et al. [106] determined the effectiveness of antiangiogenic activity of the *Quercus infectoria* acorn shell. Treatments showed that the extract possessed antiangiogenic potential, which exerts its inhibitory effect mainly through downregulation of essential mediators such as VEGF and MMPs.

### 5.4. Anti-Inflammatory and Neuroprotective Activity

Inflammation is a common pathological phenomenon respective of various diseases. The effects of *Quercus species* on anti-inflammation have been widely studied. Moreno-Jimenez et al. [95] evaluated the anti-inflammatory activity in HT-29 cells from the leaves infusion of *Q. sideroxyla, Q. durifolia*, *and Q. eduardii*. The results demonstrated that *Q. sideroxyla* decreased the levels of the inflammatory markers COX-2 and IL-8 by modulating the expression of NF-Κb. Besides, studies *in vitro* have shown that triterpenes, isolated from acorns of *Quercus serrata* var. *brevipetiolata*, inhibit nitric oxide (NO) production and other proinflammatory cytokines [44]. Moreover, lupeol isolated from white oak leaves (*Quercus resinosa, Q. grisea, Q. laeta*, *and Q. obtusata*) was evaluated for their ability to inhibit COX-1 and COX-2 enzymes by the *in vitro* colorimetric COX (ovine) inhibitor assay. In this study, lupeol from *Q. obtusata* demonstrated a differential effect to inhibit COX-2 without inhibiting COX-1 [46]. Additionally, (−)-epicatechin, procyanidin B3, and procyanidin B4 (7) obtained from the bark of *Quercus gilva Blume* presented anti-inflammatory and antioxidative potency. The three compounds showed dose-dependent inhibitory activities on the gene expression of COX-2 and IL-1*β* [80]. Studies conducted by Vázquez-Cabral et al. [107] indicate that flavonols such as quercetin glucuronide and kaempferol 3-O-glycoside are glucuronidated by the action of the kombucha consortium and that these metabolites are effective antioxidant and anti-inflammatory agents in human macrophages.

Neurodegenerative disorders are diseases that influence the nervous system, such as brain tumors, glioblastoma, epilepsy, Alzheimer's disease, and Parkinson's disease. It was reported that extracts from *Quercus suber* and *Quercus ilex* showed neuroprotective effects through inhibition of acetylcholinesterase (AChE) and butyrylcholinesterase (BChE) and protection of the human dopaminergic cell line SH-SY5Y [84]. Indeed, Gezici and Sekeroglu [43] reported that extracts from the shell, cup, and acorn parts of *Quercus coccifera* had notable AChE and BChE inhibition. Inhibitors of this enzyme are used to alleviate symptoms associated with Alzheimer's disease [84]. In another investigation, Lei et al. [45] showed potent antineuroinflammatory activity of triterpenoids isolated from Chinese acorns (*Quercus serrata* var. *brevipetiolata*), which suggested that these triterpenoids might have activities against Alzheimer's disease.

### 5.5. Hepatoprotective

Xu et al. [108] reported that acorns (*Quercus liaotungensis*) and their galloyl triterpenes exhibited stronger antiproliferative effects against t-HSC/Cl-6 cells than the reference silymarin, suggesting its potential for being developed into antihepatic fibrosis food or medicine. In the same year, Singh and Bisht [48] investigated *in vivo* hepatoprotective activity of the root extract of *Q. oblongata D. DON* and clearly showed the positive effect of the ethanolic extract at the dose of 300 mg/kg in comparison to the reference silymarin. Similarly, it was stated that the administration of 300 mg/kg of the *Q. dilatata* extract displayed protective effect against bisphenol A- (BPA-) induced hepatotoxicity by restoring hepatic inflammation towards normal [109]. Other studies performed by Toori et al. [110] investigated the hepatoprotective effects of acorn extracts on carbon tetrachloride-induced liver damage in rats. Their analyses showed that the aqueous extract at 250 and 500 mg/kg displayed excellent hepatoprotective potential, indicating that this solvent is a better alternative, with no toxic effects. In addition, several studies have shown that *Quercus* spp. exhibited hepatoprotective effects [111].

### 5.6. Antidiabetic Effect

Diabetes is a chronic disease characterized by high blood glucose levels that result from the body's inability to produce insulin [112]. One promising approach for the management of diabetes is to postpone the absorption of glucose by inhibiting carbohydrate-hydrolyzing enzymes (*α*-amylase and *α*-glucosidase) [85].

Custódio et al. [84] studied the inhibitory effect of leaves and acorns from *Quercus suber* on key enzymes relevant for hyperglycemia (*α*-amylase and *α*-glucosidase). Their study showed that the best results were obtained with the water and methanol leaves extracts with values of 97 and 89%, respectively, which could most likely be attributed to their higher phenolic content. Moreover, the extract from the bark of *Q. coccifera* exhibited stronger *α*-glucosidase inhibitory activity with an IC50 value of 3.26 ± 0.08 *µ*g/mL than that reported for acarbose IC50: 50.45 ± 0.20 *µ*g/ml [83]. In another work, triterpenoids obtained from acorns of *Quercus liaotungensis* have been studied for their inhibitory activity against *α*-glucosidase, *α*-amylase, and protein-tyrosine phosphatase 1B. The authors found that all the compounds showed strong inhibitory effects on PTP1B and *α*-glucosidase, but inhibition of *α*-amylase was not observed [87]. It was also reported that the polyphenol fraction from acorn leaves (*Quercus liaotungensis*) inhibited *α*-glucosidase and PTP1B activity [49]. Similarly, Yin et al. [85] reported that *Mongolian oak* cups might be a source of ellagic acid (EA), which possess prominent inhibitory activities against *α*-glucosidase, *α*-amylase, and formation of AGEs. Other authors reported that the chloroform extract from *Quercus dilatata* exhibited maximum antidiabetic activity *α*-amylase inhibition of 21.61 ± 1.53% at 200 *μ*g/ml [22]. In addition, condensed tannin fractions isolated from the leaves of *Quercus phillyraeoides* presented potent *α*-glucosidase inhibitory activities with IC50 values in the range of 2.60 to 3.14 *µ*g/ml, respectively [91].

Furthermore, Lin et al. [81] established that hydrolysable tannins are responsible for the lower digestibility of the acorn of *Quercus fabrei Hance*. These results further support the potential use of the acorn for preparation of low glycemic index foods. In a particular study, Ahmadi et al. [113] reported that prebiotics from the acorn can ameliorate HFD-induced defects in the glucose metabolism via positive modulation of the gut-microbiome-brain axis.

Gamboa-Gómez et al. [114] showed *in vitro* and *in vivo* antihyperglycemic and antioxidant effects of oak leaves infusions and fermented beverages from *Quercus convallata* and *Q. arizonica* using female C57BL/6 mice. Their results indicated that oak leaves infusions and fermented beverages exhibited exerted inhibition of *α*-amylase (8–15% and 5–9%, respectively) and *α*-glucosidase (98% and 99%, respectively).

Overall, *Quercus* species may serve as an alternative source of potential antidiabetic agents, and more analysis in *in vivo* and clinical studies is required to validate these *in vitro* findings.

### 5.7. Skin Disorder

Melanin is mainly responsible for skin and hair colors. It plays an important role in protecting the skin against the harmful effects of UV radiation. However, an excessive accumulation of melanin creates various skin dermatological disorders like irregular skin hyperpigmentation and aesthetic problems [115]. Tyrosinase is a key enzyme in melanin biosynthesis. Inhibition of this enzyme decreases melanin production and deposition [116].

Sari et al. [83] showed that polydatin isolated from the *Quercus coccifera* bark displayed potent tyrosinase inhibition compared to the positive control kojic acid, with an IC50 value of 4.05 ± 0.30 *µ*g/ml. In addition, Kim et al. [117] analyzed the effect of some polyamine derivatives from the bee pollen extract of *Q. mongolica* against the tyrosinase enzyme. They observed that polyamine derivatives with coumaroyl and caffeoyl moieties exhibited higher tyrosinase inhibitory potential than the others isolated with IC50 values of 19.5–85.8 Μm. Lee and coworkers [118] explored the antidermatitis effects of *oak wood vinegar* (OWV) in the DNCB-induced contact dermatitis mice model and showed that OWV has anti-inflammatory and antiproliferative activity in a DNCB-induced contact dermatitis mice model. This activity may be linked to STAT3 inactivation.

The extract from *Quercus suber* leaves showed effectiveness in the prevention of photo-induced oxidative stress in the skin through scavenging multiple ROS and RNS [119]. Moreover, Koseki et al. [120] showed that the extract of *Quercus acutissima Cortex* inhibited androgen-related pathogenesis of acne, testosterone conversion, and sebum synthesis, partially via 5*α*-reductase inhibition.

In consequence, it is possible to conclude that the *Quercus* species can be an important ingredient in the cosmetic product.

## 6. Conclusion

For a long time, *Quercus* species have been used as a traditional medicine in various countries and tribes. The bark, fruit, and leaves of the species from the genus were reported to possess a broad spectrum of biological effects, such as antioxidant, antidiabetic, anticancer, anti-inflammatory, and antibacterial. The current phytochemical studies of the species from the genus *Quercus* showed that phenolic acids (particularly gallic and ellagic acids and their derivatives), flavonoids (particularly flavan-3-ol), and tannins are somehow ubiquitous in all *Quercus* species. From these researches, phenolic compounds, triterpenoids, and flavonoids have a positive effect on anti-inflammatory, antidiabetic, and anticancer actions which can be considered as promising candidates for the development of novel pharmaceutical agents. For this, additional research on other *Quercus* species need much attention from biochemists for studying their detailed chemical profile and health effect, and also more studies are required to evaluate the safety, side effect, and efficacy of extracts.

## Figures and Tables

**Table 1:** Medicinal uses of some *Quercus* species.

Species	Part	Medicinal use	Reference
*Quercus alba L.*	Bark	Used as astringent, venotonic, and hemostatic	[7]
*Quercus acutissima Carr*	Acorn	Treat colitis, stomatitis, labor pains, obesity laryngopharyngitis diseases, astrictiona, diarrheaa, and furuncles	[8, 9]
*Quercus brantii Lindl*	Leaves Bark Acorn Gall	Astringent effects, treatment of tonsillitis, and throat infections Chronic skin diseases such as eczema and varicose veins Diarrhea, internal enzymes, indigestion, stomach pain, anemia, rickets, and tuberculosis Stomach tonic, astringent, and bleeding stopper Diarrhea, coughing, mouth ulcer, and stomach ulcer	[10–13]
*Quercus cerris L.*	Acorn Bark	Beverage, throat inflammation, cicatrizing for wounds of livestock, tea for female disorders, ointment for wounds, diaphoretic, hemorrhoids, intestinal inflammation, psoriasis, thinness, and fodder	[14–18]
*Quercus coccifera L.*	Leaves Gall Acorn	Fodder, wild vegetables, astringent enuresis, metritis, gingivitis, dermatitis, diarrhea, vaginal diseases, cough, and hypertension	[19–21]
*Quercus dilatata*	Acorn Leaves Bark Wood Flowers	Serve as brain, sexual tonic, cleaning teeth eradication of gonorrhea, urinary tract infections in district Swat Sore mouth throat in Lawat district astringent, diuretic, diarrhea, indigestion and asthma in Poonch Valley, clean foul sores Treatment of diarrhea, menorrhagia, and gastrointestinal	[22–24]
*Quercus dentata Thunb*	Gall	Dysentery, diarrheaa	[8]
*Quercus ilex L.*	Gall Roasted seeds	Used as aesthetic hair, gingiva, tonic drink coffee, fodder	[25, 26]
*Quercus incana Roxb.*	Leaves Bark Acorn Wood	Used as astringent diuretic, antidiarrheal agent, treatment asthma. Antipyretic, antirheumatism, antidiabetic, and antiarthritic, gastrointestinal disorders, inflammations of the oral, genital, anal mucosa inflammation of the skin, skeletomuscular problems and antiarthritic purposes	[27–29]
*Quercus infectoria-Olivier.*	Gall	Used as astringent, diabetes disease, restore the elasticity of the uterine wall, inflammatory disorders, wound infections after childbirth, treatment of toothache, gingivitis, skin disorder, antiseptic, antistomatitis, deodorant, derivative, desiccant, expectorant styptic, tonic, tonic to teeth and wound healing	[18, 30–32]
*Quercus leucotrichophora A. Camus.*	Acorn Leaves Bark	Treatment of urinary infection, cure toothache and piles, astringent, diarrhea, stomach ache cure, gonorrhea, asthma, hemorrhages, dysentery, gonorrheal digestive disorders, stomach pain, diuretic, urinary disorder, snake bite, check dysentery	[33–35]
*Quercus robur L*.	Leaves Bark	Diabetes, diarrhea	[36]

**Table 2:** Polyphenolic compounds in *Quercus* species.

Compound isolated	Chemical structure	Species	Reference
Gallic acid	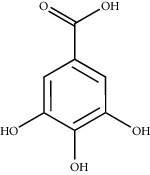	*Q. acuta* ^*(1)*^ *Q. alba* ^*(7)*^ *Q. arizonica* ^*(3)*^ *Q. convallata* ^*(3)*^ *Q. durifolia* ^*(3)*^ *Q. faginea* ^*(7)*^ *Q. eduardii* ^*(3)*^ *Q. glauca* ^*(1)*^ *Q. ilex* ^*(4)*^ *Q. humboldtii* ^*(5)*^ *Q. myrsinaefolia* ^*(1)*^ *Q. petraea* ^*(7)*^ *Q. phillyraeoides* ^*(1)*^ *Q. pyrenaica* ^*(7)*^ *Q. robur* ^*(7)*^ *Q. resinosa* ^*(2,3)*^ *Q. rotundifolia* ^*(1)*^ *Q. salicina* ^*(1)*^ *Q. stenophylla* ^*(4)*^ *Q. suber* ^*(1,7)*^ *Q. sideroxyla* ^*(4)*^ *Q.* spp. ^*(7)*^	(1) [67] (2) [47] (3) [68] (4) [69] (5) [70] (6) [71] (7) [72]
Quinic acid	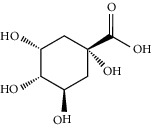	*Q. suber*	[73]
Gentisic acid	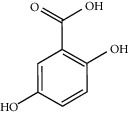	*Q. acuta* ^*(1)*^ *Q. glauca* ^*(1)*^ *Q. phillyraeoides* ^*(1)*^ *Q. salicina* ^*(1)*^ *Q.* spp. ^*(2)*^	(1) [67] (2) [72]
Chlorogenic acid	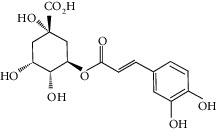	*Q. acuta* ^*(1)*^ *Q. myrsinaefolia* ^*(1)*^ *Q. phillyraeoides* ^*(1)*^ *Q. resinosa* ^*(2)*^ *Q. salicina* ^*(1)*^ *Q. mohriana* ^*(4)*^ *Q. muhlenbergii* ^*(4)*^ *Q. oblongifolia* ^*(4)*^ *Q. pungens* ^*(4)*^ *Q. turbinella* ^*(4)*^ *Q. emoryi* ^*(4)*^ *Q. hypoleucoides* ^*(4)*^ *Q. suber* ^*(3)*^	(1) [67] (2) [47] (3) [71] (4) [74]
Caffeic acid	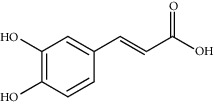	*Q. acuta* ^*(1)*^ *Q. myrsinaefolia* ^*(1)*^ *Q. phillyraeoides* ^*(1)*^ *Q. resinosa* ^*(2)*^ *Q. robur* ^*(1)*^ *Q. salicina* ^*(1)*^ *Q.* spp. ^*(3)*^	(1) [67] (2) [47] (3) [72]
Ferulic acid	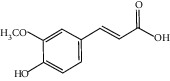	*Q. acuta (1)* *Q. faginea (2)* *Q. glauca (1)* *Q. myrsinaefolia (1)* *Q. petraea (2)* *Q. phillyraeoides (1)* *Q. petraea (2)* *Q. pyrenaica (2)* *Q. robur (2)* *Q. salicina (1)* *Q. suber (3)*	(1) [67] (2) [72] (3) [75]
Vanillic acid	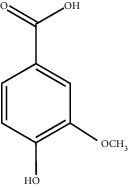	*Q. acuta (1)* *Q. alba (2)* *Q. faginea (2)* *Q. glauca (1)* *Q. humboldtii (4)* *Q. myrsinaefolia (1)* *Q. petraea (2)* *Q. phillyraeoides (2)* *Q. pyrenaica (2)* *Q. resinosa (2)* *Q. robur (2)* *Q. salicina (1)* *Q. suber (3)* *Q.* spp. *(2)*	(1) [67] (2) [72] (3) [75] (4) [70]
Homogentisic acid	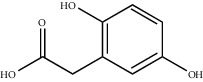	*Q. acuta* *Q. myrsinaefolia* *Q. phillyraeoides* *Q. salicina*	[67]
Protocatechuic acid	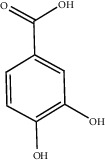	*Q. acuta* ^*(1)*^ *Q. glauca* ^*(1)*^ *Q. myrsinaefolia* ^*(1)*^ *Q. phillyraeoides* ^*(1)*^ *Q. resinosa* ^*(2)*^ *Q. salicina* ^*(1)*^ *Q. suber* ^*(3,4,5)*^ *Q.* spp. ^*(6)*^	(1) [67] (2) [47] (3) [71] (4) [75] (5) [76] (6) [72]
Syringic acid	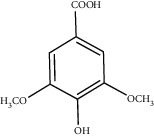	*Q. acuta* ^*(1)*^ *Q. alba* ^*(3,4)*^ *Q. faginea* ^*(3)*^ *Q. glauca* ^*(1)*^ *Q. myrsinaefolia* ^*(1)*^ *Q. petraea* ^*(3)*^ *Q. phillyraeoides* ^*(1)*^ *Q. pyrenaica* ^*(3)*^ *Q. resinosa* ^*(2)*^ *Q. robur* ^*(3)*^ *Q. salicina* ^*(1)*^ *Q. humboldtii* ^*(4)*^ *Q. petraea* ^*(4)*^ *Q.* spp. ^*(3)*^	(1) [67] (2) [47] (3) [72] (4) [70]
Galloylquinic acid	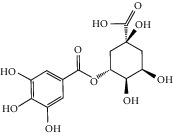	*Q. emoryi* *Q. hypoleucoides*	[74]
Vanillin	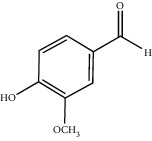	*Q. acuta* ^*(1)*^ *Q. alba* ^*(3)*^ *Q. faginea* ^*(3)*^ *Q. glauca* ^*(1)*^ *Q. myrsinaefolia* ^*(1)*^ *Q. petraea* ^*(3)*^ *Q. pyrenaica* ^*(3)*^ *Q. resinosa* ^*(2)*^ *Q. robur* ^*(3)*^ *Q. salicina* ^*(1)*^ *Q. suber* ^*(4,5,6)*^ *Q.* spp. ^*(3)*^	(1) [67] (2) [47] (3) [72] (4) [75] (5) [73] (6) [71]
Kaempferol	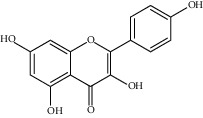	*Q. acutaQ. glauca* ^*(1)*^ *Q. myrsinaefolia* ^*(1)*^ *Q. phillyraeoides* ^*(1)*^ *Q. salicina* ^*(1)*^ *Q. stenophylla* ^*(2)*^	(1) [67] (2) [77]
Quercetin	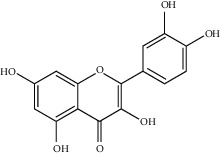	*Q. incana* ^*(1)*^ *Q. glauca* ^*(2)*^ *Q. myrsinaefolia* ^*(2)*^ *Q. phillyraeoides* ^*(2)*^ *Q. salicina* ^*(2)*^	(1) [78] (2) [67]
Naringenin	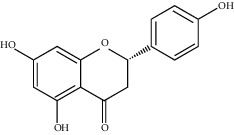	*Q. acuta* ^*(1)*^ *Q. glauca* ^*(1)*^ *Q. myrsinaefolia* ^*(1)*^ *Q. phillyraeoides* ^*(1)*^ *Q. salicina* ^*(1)*^ *Q. suber* ^*(2)*^	(1) [67] (2) [73]
Acutissimin A	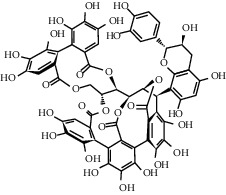	*Q. arizonica* *Q. gambelii* *Q. grisea* *Q. havardii* *Q. mohriana* *Q. muhlenbergii* *Q. oblongifolia* *Q. pungens* *Q. rugosa* *Q. turbinella*	[74]
Epicatechin	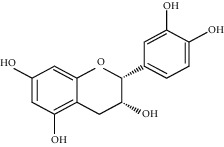	*Q. convallata* *Q. resinosa* *Q. gilva*	[47,79,80]
Castalagin	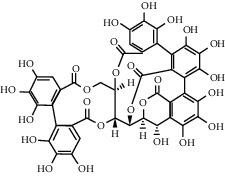	*Q. arizonica* ^*(1)*^ *Q. fabri* ^*(4)*^ *Q. gambelii* ^*(1)*^ *Q. grisea* ^*(1)*^ *Q. havardii* ^*(1)*^ *Q. mohriana* ^*(1)*^ *Q. muhlenbergii* ^*(1)*^ *Q. oblongifolia* ^*(1)*^ *Q. pungens* ^*(1)*^ *Q. rugosa* ^*(1)*^ *Q. turbinella* ^*(1)*^ *Q. suber* ^*(2,3)*^	(1) [74] (2) [71] (3) [16] (4) [81]
Gallocatechin	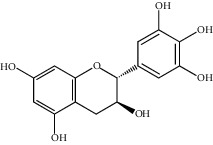	*Q. grisea* ^*(2)*^ *Q. resinosa* ^*(1,2)*^ *Q. arizonica* ^*(2)*^ *Q. convallata* ^*(2)*^ *Q. durifolia* ^*(2)*^ *Q. eduardii* ^*(2)*^ *Q. sideroxyla* ^*(2)*^	(1) [47] (2) [68]
Epicatechin gallate	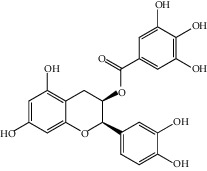	*Q. arizonica* ^*(1)*^ *Q. resinosa* ^*(1)*^ *Q. eduardii* ^*(2)*^ *Q. convallat* ^*(1)*^ *Q. ilex* ^*(2)*^ *Q. gilva* ^*(3)*^ *Q. sideroxyla* ^*(1)*^	(1) [68] (2) [82] (3) [80]
Epigallocatechin-3-gallate	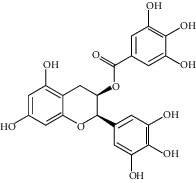	*Q. ilex* ^*(1)*^ *Q. grisea* ^*(2)*^ *Q. resinosa* ^*(2)*^ *Q. arizonica* ^*(2)*^ *Q. convallata* ^*(2)*^	(1) [82] (2) [68]
Eupatorin	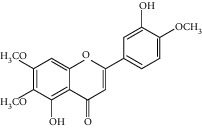	*Q. incana*	[29]
Cirsimaritin	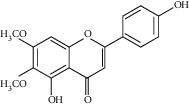	*Q. incana*	[29]
Methyl gallate	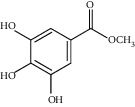	*Q. incana*	[78]
Picraquassioside D	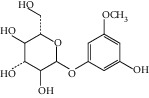		
Quercussioside	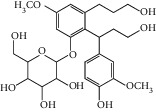		
Procyanidin B3	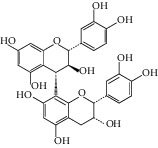		
Procyanidin B4	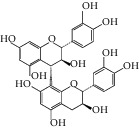	*Q. gilva*	[80]
Quercuschin	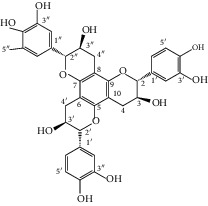	*Q. incana*	[78]
Lyoniresinol-9-O-*β*-xylopyranoside	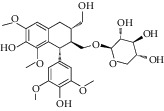	*Q. coccifera* *Q. gilva*	[80,83]
Polydatin	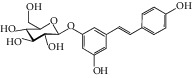	*Q. coccifera*	[83]

**Table 3:** Biological activities of some compounds from *Quercus* species.

No	Compound name	Biological activity	Description	Reference
1	Polydatin	Dermatological disorders	Showed potent tyrosinase inhibition compared to the positive control, kojic acid, with an IC50 value of 4.05 ± 0.30 *µ*g/mL	[83]
2	(-)-8-Chlorocatechin	Antidiabetic	The most potent isolate, also more potent than the positive control, acarbose, with an IC50 value of 43.60 ± 0.67 *µ*g/mL	[83]
3	*β*-Sitosterol-D-glucoside		Showed moderate inhibitory activity against *α*-glucosidase	
4	Tiliroside	Antidiabetic	Highest *α*-glucosidase inhibitory activity with an IC50 of 28.36 ± 0.11 mmol/L	[86, 91]
5	Epicatechin	Antioxidant	Higher antioxidant activity with inhibitory concentrations (IC50) of 22.55 ± 2.23 mmol/L than that of quercetin, which was used as the standard, with an IC50 of 28.08 ± 2.39 mmol/L	[86]
6	**A**-3-O-Galloyloleanolic acid-23-acetoxy-3-O-galloyloleanolic acid **B**-3-Acetoxy-23-O-galloyloleanolic acid **C**-3-O-Galloylursolic acid	Antidiabetic	Most of the compounds showed strong inhibitory effects on PTP1B and *α*-glucosidase, their IC50 values were about 6-fold to 20-fold lower than positive control	[49]
7	Quercetin-3-O-(2″-O-galloyl)-*β*-galactopyranoside	Antidiabetic	Increased the survival of pancreatic beta cells by reducing the production of reactive oxygen species and enhancing the activities of superoxide dismutase, catalase, and glutathione in MIN6 cells damaged by H_2_O_2_. The preliminary mechanism by which the compound protects pancreatic beta cells was through the nuclear factor erythroid-2-related factor 2 (Nrf_2_)/heme oxygenase-1 HO-1 pathway	[87]
8	Betulin	Anticancer	Treatment with betulin increases expression of the caspase family (i.e., caspase-3, -6, and -9), proapoptotic genes (BAX and BAK), and inhibiting antiapoptotic genes (BCL-2L1 and p53) and could also regulate metastasis by inhibiting MMP-2/-	[29]
9	Roburgenic acid	Cytotoxicity	Roburgenic acid was the most active compound (IC50 = 19.7 *μ*M) reaching a comparable value to those of positive controls	[90]
10	3-O-Galloyloleanolic acid	Antihepatic fibrosis and antioxidant	Upregulated the expression levels of Nrf_2_ and HO-1 in t-HSC/Cl-6 cells	[49]
11	**A**-23-O-Galloyl-2*α*,3*β*,19*α*-trihydroxyurs-12-en- 28-oic acid 28-O-*β*-D-glucopyranoside **B**-3-O-Galloyl-2*α*,19*α*,23 trihydroxyurs-12-en-28-oic acid 28-O-*β*-D-glucopyranoside **C**-3,23-O-Butylidene 2*α*,3*β*,19*α*,23 tetrahydroxyolean-12-en-28-oic acid 28-O-*β*-D-glucopyranoside **D**-2*α*,3*β*,23-Trihydroxylup-20(29)-en-28-oic acid 28- O-*β*-D-glucopyranoside	Antineuroinflammatory	The compounds reduce dose dependently the expression levels of proinflammatory mediator iNOS and reduce the COX-2 expression induced by LPS in BV-2 cells	[45]
12	**A**-3,23-O-Methyl butyrate 2,3,19,23-tetrahydroxy-urs-12-en-28-oic acid -d glucopyranosyl ester **B**-3,23-O-Methyl butyrate-2,3,19,23-tetrahydroxy-olean-12-en-28-oic acid -d-glucopyranosyl ester **C**-23-Acetoxy-2,3,19 trihydroxyurs-12-en-28-oic acid	Anti-inflammatory	The three compounds showed pronounced anti-inflammatory activities compared to positive control indomethacin (IC50 (*µ*M): 8.2 ± 0.6, 12.8 ± 0.8, 19.1 ± 6.1, and 47.4 ± 4.5, respectively) and higher activity against proinflammatory cytokines (IL-6 and IL-8)	[44]
13	**A**-(-)-Epicatechin **B**-Procyanidin B3 **C**-Procyanidin B4	Anti-inflammatory	The three compounds showed dose-dependent inhibitory activities on gene expression of COX-2 and IL-1*β*	[80]

## Abbreviations

ATCC: American Type Culture Collection
ABTS: 2,2′-Azino-bis(3-ethylbenzothiazoline-6-sulphonic acid)
BAX: Bcl-2-associated X protein
BAK: Bcl-2 antagonist/killer 1
BHT: Butylated hydroxytoluene
COX-1: Cyclooxygenase-1
COX-2: Cyclooxygenase-2
DPPH: 2,2-Diphenyl-1-picrylhydrazyl
FRAP: Ferric reducing antioxidant power
HRESIMS: High-resolution electrospray ionization mass spectrometry
QIE: *Quercus infectoria* extract
Q: *Quercus*
IL: Interleukin
iNOS: Inducible nitric oxide synthase
IC50: 50% inhibiting concentration
MBC: Minimum bactericidal concentration
MMPs: Matrix metalloproteinases
MIC: Minimum inhibitory concentration
NF-k*β*: Nuclear factor kappa-light-chain-enhancer of activated B cells nitric oxide
PTP1B: Protein tyrosine phosphatase 1B
ROS: Reactive oxygen species
RNS: Reactive nitrogen species
STAT3: Signal transducer and activator of transcription 3
spp.: Plural species
UV: Ultraviolet
VEGF: Vascular endothelial growth factor.

## Additional Points

(i) The medicinal uses of *Quercus* species are reviewed. (ii) Phenolic acids, flavonoids, and tannins are somehow ubiquitous in all *Quercus* species. (iii) Triterpenoids have a positive effect on anti-inflammatory and cytotoxic activity. (iv) The pharmacological aspect of extracts and pure compounds is reported.
